# Task-shifting in dementia care: a comparative analysis of consultation models and proposed collaborative ecosystem in Japan

**DOI:** 10.3389/fpsyt.2025.1504753

**Published:** 2025-06-13

**Authors:** Kae Ito, Tsuyoshi Okamura

**Affiliations:** Tokyo Metropolitan Institute for Geriatrics and Gerontology, Itabashi-ku, Tokyo, Japan

**Keywords:** dementia, psychiatric outreach service, psychogeriatric outreach service, primary care physician, community care, task-shift

## Abstract

**Introduction:**

With an aging population, task-shifting in dementia care has been proposed to meet the needs of persons living with dementia (PLWD). In Japan, the Initial-phase Intensive Support Service (IPIS), led by primary care physicians, was introduced alongside traditional Psychogeriatric Services (PS), which are psychiatrist-led. However, the impact of this shift on care capacity remains unclear.

**Methods:**

This study employed a convergent mixed-methods design to examine two dementia outreach services in Tokyo: PS and IPIS. This study aimed to: 1) compare the content of consultations between PS and IPIS; 2) identify tasks that could not be shifted; and 3) propose a collaborative care model to address identified service gaps. We analyzed 121 PS cases and 213 IPIS case records, and conducted interviews with team members to explore cases where IPIS was perceived as less equipped.

**Results:**

The IPIS Service was generally suitable for early-stage dementia without severe behavioral symptoms but less equipped to manage: 1) mental health conditions beyond dementia; 2) long-standing psychosocial issues; and 3) acute crises—areas where PS traditionally intervened.

**Discussion:**

The findings highlight the need for strengthened collaboration between primary care and psychiatric services. We propose a collaborative care ecosystem in which primary care physicians lead community-based dementia care, supported by psychogeriatric consultation.

## 1 Introduction

Japan has the highest aging rate in the world, which has been increasing rapidly since around 1970 and surpassed 29% in recent years (1). A distinctive feature of Japan’s aging population is the rapid growth of the “super-old” group—those aged 85 and above (2). Among this population, the prevalence of dementia is estimated to be as high as 40–80% (3). As a result, Japan is now facing an unprecedented number of persons living with dementia (PLWD).

Given this context, the number of individuals with dementia is projected to far exceed the capacity of specialized dementia care providers, such as geriatric psychiatrists and neurologists. Meanwhile, primary healthcare services remain widely available and accessible in communities. Primary care physicians are therefore expected to play a broader role in dementia care, from early recognition of cognitive decline to end-of-life support (4). A recent nationwide study in China also highlights both the potential and the challenges of primary care involvement in dementia care, pointing to gaps in training and the need for stronger support systems (5).

The growing demand on healthcare systems has led to global discussions about task-shifting in dementia care. The 2016 World Alzheimer Report recommended a “task-shifted and task-shared healthcare model” to optimize resources and improve care delivery (6). In this study, we adopt Fulton’s operational definition of task shifting as “delegating selected tasks to existing or new cadres with either less training or narrowly tailored training” (7). For example, Alzheimer’s Disease International describes the shift of responsibilities from highly specialized professionals, such as neurologists, to primary care physicians (6).

In parallel, the global mental health field increasingly supports a shift from long-term institutionalization toward community-based services (8, 9). Within this framework, outreach psychiatric services have emerged as critical for reaching individuals who are unable or unwilling to visit conventional mental health clinics (10). These individuals often require both medical and psychosocial support, typically delivered by multidisciplinary teams (11). With the aging of populations worldwide, these outreach services are increasingly used by older individuals. In Japan, a 2013 report showed that half of outreach service users were over the age of 50 (12), although detailed data on the psychiatric needs of older persons remain limited.

In response to these challenges, the Japanese government launched the Initial-phase Intensive Support Service for Dementia (IPIS Service) in 2013, alongside traditional Psychogeriatric Services (PS). This initiative specifically targets PLWD and aims to promote timely engagement and support (13). It aligns with Japan’s national dementia policy, which emphasizes deinstitutionalization and aims to ensure that essential services are accessible within 30 minutes by 2025, when the baby boomer generation reaches age 75. By 2019, it became mandatory for each local government to establish at least one Initial-phase Intensive Support Team (IPIST) (14), and this goal has largely been achieved.

**Figure 1:**
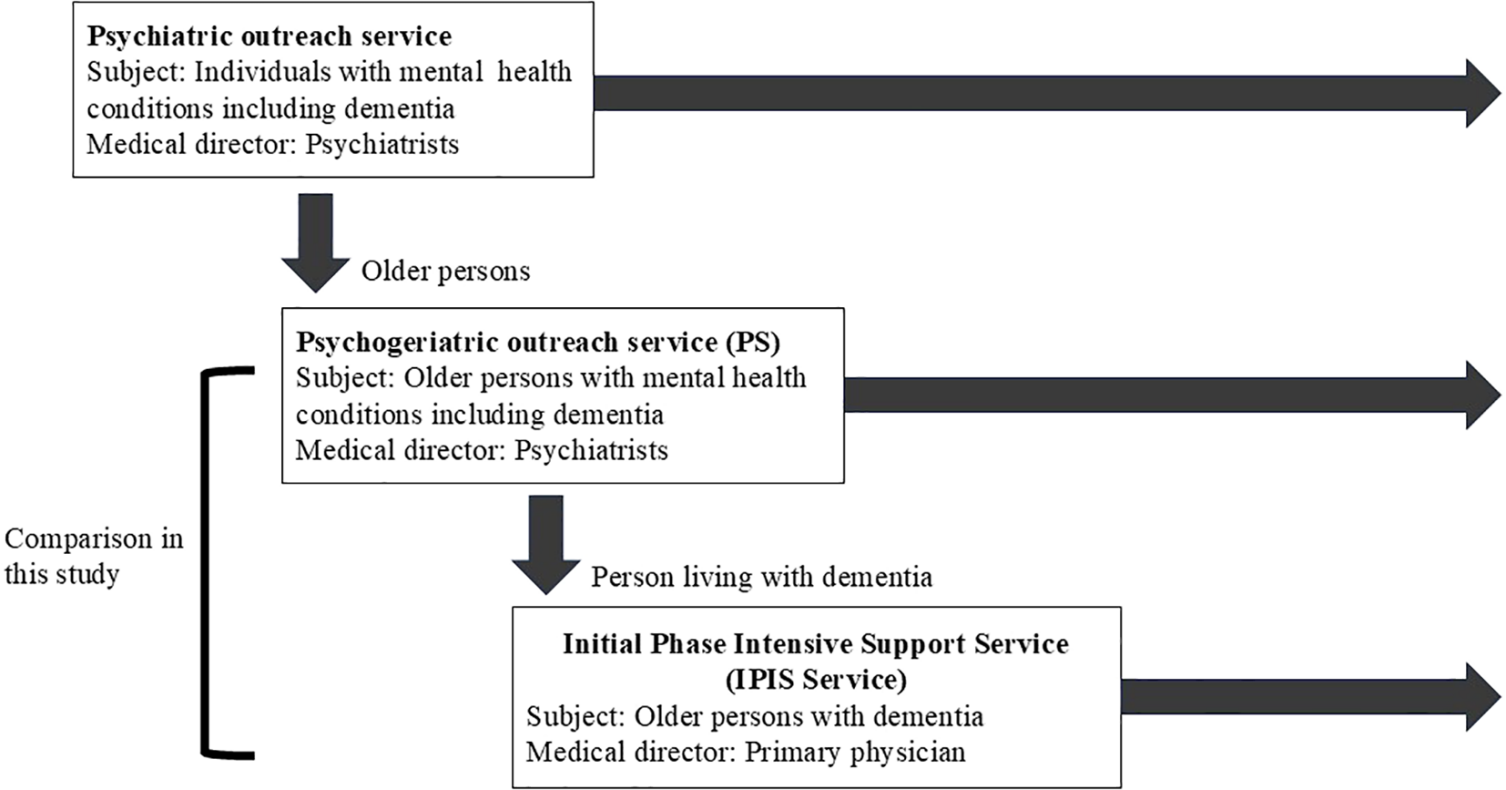
Context of Japanese psychiatric outreach team’s response to social change. Psychiatric outreach services traditionally included older persons. A psychogeriatric outreach service was introduced, separating older persons from general services. Later, the Initial-phase Intensive Support Service for Dementia was established to specifically support persons living with dementia.

Since outreach services are often less resource-efficient than hospital-based care, task-shifting in dementia outreach has become a necessary strategy to meet the growing demand. **Figure 1** illustrates the structure of the Japanese psychiatric outreach system, which reflects global trends in task-shifting.

Given the exploratory nature of this study, our primary aim is to examine the processes and structural conditions that shape the functioning of the IPIS Service and its interaction with the traditional psychogeriatric service. We do not assess patient-level outcomes such as clinical improvement or quality of life, which would require future research using standardized metrics and statistical adjustments for baseline differences.

*Mechanism*: IPISTs consist of a dementia support doctor and other health professionals from Community General Support Centers (CGSCs). All dementia support doctors have completed a two-day educational program. Of these, 85.9% are not specialists in dementia care, with 53.8% being primary care physicians. Regarding specialties, 49.3% are internal medicine specialists, a rate 2.8 times higher than psychiatrists (17.6%) (15). This uneven distribution highlights the challenge of placing a psychiatrist in every IPIST.

The IPIS Services support community-dwelling individuals with suspected or diagnosed dementia who lack appropriate care or present with severe behavioral symptoms (16). However, referrals to IPIS are not mandatory, which has kept consultation requests to the PS consistently high.

The aims of the present study were to 1) compare the content of consultations between the Psychogeriatric Service (PS) and the IPIS Service following the task-shift; and 2) identify tasks that could not be shifted; and 3) propose a collaborative care ecosystem to address these gaps.

## 2 Material and methods

### 2.1 Methodology

This study used a convergent mixed-method design (17), grounded in a realist approach, to examine how the new primary care physician-led team for PLWD operates and the need for collaboration with the traditional psychogeriatric team to build an integrated care ecosystem. Insights from team members of both services clarified current challenges. The context and mechanism are discussed in the introduction, and the service process are drawn from outreach records and service providers’ narratives. This study focuses on examining service processes rather than evaluating outcome effectiveness.

In the convergent mixed-methods design, quantitative and qualitative data were collected in parallel and given equal priority. Quantitative data (objective service records) and qualitative data (interviews and FGIs with service providers) were analyzed separately, and the findings from each strand were then integrated during the interpretation of results. This approach allowed the quantitative results to be contextualized and enriched by the qualitative insights, and vice versa, thereby providing a comprehensive understanding of the research questions. A flow diagram illustrating the mixed-methods process and data flow is provided in **Supplementary Figure 1**.

### 2.2 Setting

The study focused on two municipal outreach services in Tokyo’s Itabashi District, targeting older individuals with psychiatric symptoms. Itabashi has a population of 570,000, with 23% aged 65 years or older (September 2024). The services examined were the PS, launched in 1986, and the IPIS Service. The PS team included a geriatric psychiatrist and municipal staff, addressing older persons with complex health and social care needs (18).

### 2.3 Data collection

For this convergent mixed-methods study, data collection encompassed two complementary components: objective quantitative data from service records and subjective qualitative data from interviews.

#### 2.3.1 Objective data

A consecutive case series study was conducted using the records of 121 cases eligible for PS from April 2016 to March 2022, covering the period following the introduction of the IPIS Service. In addition, the records of 213 cases eligible for the IPIS Service were collected from April 2016 to March 2021, corresponding to the period since the service was initiated.

The following data were collected from the case records.

*Basic variables.*

• Sociodemographic characteristics of age, sex, marital status, living condition, source of income, and years of education.

*Clinical assessments.*

• Psychiatric diagnosis: Diagnosis according to the ICD-10 Classification of Mental and Behavioral Disorders (19).

• Clinical stage of dementia: The clinical stage of dementia was assessed according to the Japanese version of the Clinical Dementia Rating Scale (CDR) (20, 21).

• Basic activities of daily living (BADL): BADL were assessed at three levels: independent, requires partial assistance, and requires full assistance for walking, toileting, bathing, and eating, respectively. For the overall assessment of BADL, partial assistance was defined as requiring assistance on ≤3 of the four items, and full assistance as requiring assistance for all four items.

The first author conducted assessments of individuals in the PS group, and IPIST members conducted assessments of the IPIS Service group.

*Service utilization.*

Data were collected on the use of medical services, long-term care insurance services, and rights protection services.

*Issues faced by the individuals from the PS and IPIS Service.*

Issues were collected based on the analytical framework developed in our previous study (**Table 1**) (18).

**Table 1:** Analytical frame of complex care needs of older persons, comparison of Psychogeriatric Service and Initial Phase Intensive Support Service, and number of participants who assessed the IPIS Service as ineffective for cases with the below characteristics.

Number of Cases with Care Needs per Category/Subcategory																		Number of participants who indicated that cases with issues in that category were difficult for IPIS Service to manage.						
		PS Group		IPIS Service Group		Chi- squared	p -value			PS Group		IPIS Service Group		Chi- squared	p - value	Total		CGSCs	MSs of IPISTs	MSs of PS team	DSDs	Psys
		n	%	n	%					n	%	n	%			n	%	n	n	n	n	n
Total number		121		213						121		213				36		17	3	3	11	2
Category									Subcategory															
A	Mental Health Issues	94	77.7%	179	84.0%	2.0	0.153	A1	Undetected dementia	68	56.2%	148	69.5%	5.9	0.015*	1	27.8%	0	0	0	1	0
								A2	Untreated BPSD	16	13.2%	58	27.2%	9.3	0.002*	15	41.7%	4	3	0	8	0
								A3	Undetected mental health conditions beyond dementia	24	19.8%	11	5.2%	16.9	<0.001*	31	86.1%	15	6	0	8	2
								A4	Acute phase of mental health conditions	6	5.0%	0	0.0%	12.4	<0.001*	32	88.9%	15	6	0	9	2
B	Physical Health Issues	44	36.4%	62	29.1%	1.9	0.173	B1	Neglect of physical state	28	23.1%	62	29.1%	1.4	0.234	10	27.8%	8	0	1	1	0
								B2	Delirium	12	9.9%	0	0.0%	19.1	<0.001*	1	2.8%	1	0		0	0
								B3	Having trouble with hospital staff	18	14.9%	0	0.0%	38.4	<0.001*	16	44.4%	4	3	1	6	2
								B4	Issues related to end-of-life care	3	2.5%	0	0.0%	6.1	0.013*	8	22.2%	8	0		0	0
C	Family Issues	93	76.9%	88	41.3%	40.9	<0.001*	C1	Family structure with no potential caregiver	45	37.2%	57	26.8%	3.9	0.048*	19	52.8%	10	3	1	5	0
								C2	Caregiver has mental health condition	35	28.9%	28	13.1%	12.1	<0.001*	21	58.3%	10	3	3	4	1
								C3	Interference with service use by family members	10	8.3%	13	6.1%	0.6	0.459	20	55.6%	9	3	1	7	0
								C4	Abuse	36	29.8%	0	0.0%	81.0	<0.001*	32	88.9%	17	3	3	7	2
D	Issues of Neighborhood Communication	47	38.8%	53	24.9%	7.0	0.008*	D1	Aggressive behavior towards neighbors	8	6.6%	8	3.8%	1.3	0.249	20	55.6%	12	3	0	5	0
								D2	Exclusion from the community	30	24.8%	36	16.9%	3.0	0.085	21	58.3%	13	3	0	5	0
								D3	Severe domestic squalor	24	19.8%	24	11.3%	4.5	0.035*	19	52.8%	13	0	1	5	0
E	Financial Issues	32	26.4%	38	17.8%	3.4	0.066	E1	Money trouble	21	17.4%	24	11.3%	2.4	0.122	13	36.1%	8	0	1	4	0
								E2	Victim of fraud	11	9.1%	15	7.0%	0.4	0.506	20	55.6%	9	3	1	6	1

PS, Psychogeriatric Service; IPIS Service, Initial-phase Intensive Support Service for Dementia; IPIST, Initial-phase Intensive Support Team; BPSD, Behavioral and Psychological Symptoms of Dementia; CGSC, Community General Support Center; MS, municipal staff; DSD, dementia support physician; Psy, geriatric psychiatrist.*:p<0.05.

#### 2.3.2 Subjective data

##### 2.3.2.1 Narrative data

Since outcome data were not available in the case records, face-to-face interviews and focus group interviews (FGIs) were conducted with members of both service teams. The primary question asked to deepen understanding was: *What are the characteristics of cases that IPIS Service had difficulty managing, and for which another service would have been a more appropriate option?* In both FGIs and individual interviews, a semi-structured interview guide was used to ensure key topics were consistently covered and explored in depth. This guide included follow-up prompts encouraging participants to provide concrete examples and reflections related to the central question. For instance, participants were asked to describe specific challenging cases and to discuss the factors that made those cases difficult for the IPIS Service team to manage. They were also asked whether an alternative service (such as the PS or other community resources) might have been more appropriate for those cases, and to explain their reasoning. Sample questions from the guide included:

- ”Can you describe a recent case that the IPIS Service team found particularly challenging to manage? What made this case difficult?”
- ”What factors or circumstances do you believe contributed to the difficulty in managing that case?”
- ”Do you think another existing service would have been more suitable for this case? If yes, which service and why?”

These open-ended questions facilitated rich, detailed discussions and ensured consistency in data collection across the FGIs and interviews.

After obtaining informed consent, FGIs were conducted at each CGSC, and individual semi-structured interviews were conducted with dementia support doctors and municipal staff. The interviews took place between September 5, 2023 and January 5, 2024. To ensure confidentiality, the interviews were not recorded; instead, research notes were taken to document the discussions.

### 2.4 Data analysis

#### 2.4.1 Statistical analyses

Data from 121 cases eligible for PS from April 2016 to March 2022 (PS group) and 213 cases eligible for IPIS Service (IPIS Service group) were collected from April 2016 to March 2021 and analyzed as follows.

##### 2.4.1.1 Quantitative data analysis

Statistical analyses were performed using IBM SPSS Statistics version 29.0.1.0. Continuous variables were compared using the t-test, and categorical variables were assessed using the Chi-squared test to determine frequency differences. A p-value of less than 0.05 was considered significant.

##### 2.4.1.2 Qualitative data analysis

The narrative data from FGIs and interviews were analyzed using an inductive content analysis approach, guided by the predefined analytical framework shown in **Table 1**. Using this framework (developed in our previous study (18)), the research team categorized reported issues and challenges into thematic categories. For each category listed in **Table 1**, we counted the number of team members from PS and IPISTs who indicated that cases with issues in that category were difficult for IPIS Service to manage. This process enabled a descriptive quantification of qualitative responses within the framework’s categories.

##### 2.4.1.3 Integration of mixed-methods findings

After conducting the separate quantitative and qualitative analyses described above, we compared and integrated the findings from both components to draw overall conclusions. We examined how the qualitative themes corresponded to or helped explain the quantitative patterns observed in service utilization and case characteristics. Areas of convergence and divergence between the quantitative results and qualitative insights were identified, allowing the two data sources to inform and enrich each other. This integrative step provided a more nuanced understanding of the research questions than could be achieved by either component alone.

### 2.5 Rigor and trustworthiness

Throughout the study, several strategies were employed to ensure methodological rigor and enhance the trustworthiness of our findings:

- Triangulation: We utilized multiple data sources and methods by combining quantitative case record data with qualitative interview data. This methodological triangulation allowed cross-verification of findings, as patterns observed in the service records could be corroborated or further explained by the perspectives obtained from the FGIs and interviews. We also gathered input from various stakeholders (psychiatric service team members, primary care team members, and municipal staff), providing data source triangulation through differing viewpoints on the same issues.
- Member checking: To improve the credibility of the qualitative findings, the interviewer summarized key points and interpretations during the FGIs and interviews, giving participants the opportunity to confirm or clarify their statements. This immediate form of member checking ensured that the information recorded in the research notes accurately reflected participants’ perspectives. Additionally, preliminary findings were discussed with some participants informally to verify that our interpretations resonated with their experiences.
- Pre-established analytical framework: As noted, our analysis was guided by a pre-existing framework of issue categories derived from prior research (**Table 1**) (18). Using this framework in the coding process added a measure of consistency to the qualitative analysis and helped ensure that data were interpreted within a structured, evidence-based context. By applying an established classification of common issues, we enhanced the reliability of identifying themes and facilitated comparisons with findings from the earlier study.

### 2.6 Ethical considerations

All methods and procedures conducted in this study adhered to relevant guidelines and regulations. The retrospective study using data from case records of the two services was considered secondary research, using data owned by the local government agency. The use of these data was in full compliance with local government regulations. The study was approved by the Research Ethics Board of the Tokyo Metropolitan Institute of Gerontology (R21-79).

## 3 Results

### 3.1 Description of the study population

The results are shown in **Table 2**.

**Table 2:** Description of the study population.

		PS Group		IPIS Service Group						PS Group		IPIS Service Group			
		n	%	n	%	t/Chi-squared	p-value			n	%	n	%	Chi- squared	p-value
		121		213						121		213			
**Sociodemographic characteristics**									**Clinical assessments**						
Age (y)	Mean±SD	79.2± 7.4		81.6± 6.2		-3.2	<0.001*		Clinical diagnosis of psychiatric disorders					87.7	<0.001*
Sex	Male	51	42.2%	76	35.7%				Mild Cognitive Disorder	16	13.2%	2	1.8%		
	Female	70	57.9%	137	64.3%	1.4	0.243		Dementia					
Marital status	Have been married	90	74.4%	170	83.7%	4.1	0.043		AD	37	30.6%	88	79.3%		
	Never married	31	25.6%	33	16.3%				AD with CVD	16	13.2%	1	0.9%		
	Currently single	87	71.9%	144	69.2%	1.1	0.571		VaD	13	10.7%	2	1.8%		
Living condition	Living alone	61	50.4%	119	55.9%	17.9	<0.001*		Other types of dementia	5	4.1%	13	11.7%		
	Not living alone	59	49.2%	39	24.7%				Other psychiatric disorder	29	24.0%	5	4.5%		
Source of income	Old age pensions	104	86.0%	148	69.5%	39.2	<0.001*		No psychiatric disorder	5	4.1%	0	0.0%		
	Welfare	12	9.9%	22	10.3%				Clinical Dementia Rating (CDR)					23.8	<0.001*
	No income	5	4.1%	4	1.9%				0	4	3.3%	6	3.0%		
Years of education	≤9	15	28.8%	13	23.6%	0.6	0.748		0.5	26	21.5%	12	6.0%		
	10-12	23	44.2%	24	43.6%				1	53	43.8%	113	56.2%		
	≥13	14	26.9%	18	32.7%				2	35	28.9%	70	34.8%		
**Service utilization**									3	3	2.5%	0	0.0%		
Diagnosed psychiatric disorder (dementia) (+)		26(20)	21.5%	44(44)	20.7%	0.0	0.858		Basic activities of daily living (BADL)					16.1	<0.001*
Have a primary care physician		59	48.8%	129	60.6%	4.4	0.037*		Independent	43	36.5%	116	54.5%		
Long-term care insurance services utilization (+)		34	28.1%	47	22.1%	1.5	0.219		Requires partial assistance	75	62.0%	97	45.5%		
Rights advocacy program (+)		3	2.5%	0	0.0%	6.1	0.013*		Requires full assistance	3	2.5%	0	0.0%		

SD, standard deviation; AD, Alzheimer’s Disease; AD with CVD, Alzheimer’s Disease with cerebrovascular disease; VaD, Vascular dementia.*:p<0.05.

#### 3.1.1 Basic variables

Significant differences were observed between the two groups in age, living conditions, and source of income. The mean age ± standard deviation (SD) of the IPIS Service group was significantly higher than that of the PS group (p < 0.001). The frequency of individuals living alone was significantly higher in the IPIS Service group (p < 0.001). The frequency of individuals receiving welfare was significantly higher in the PS group (p < 0.001).

There were no significant differences between the two groups in the male-to-female ratio, marital status, or years of education.

#### 3.1.2 Clinical assessments

Significant differences were found in the distribution of psychiatric diagnoses, CDR, and BADL levels. The frequency of individuals diagnosed with Alzheimer’s disease was significantly higher in the IPIS Service group (p < 0.001). The frequency of individuals with CDR = 1 (mild dementia) was significantly higher in the IPIS Service group (p < 0.001).

#### 3.1.3 Service utilization

The frequencies of diagnosed dementia and use of long-term care insurance services did not differ between the two groups. However, significantly more individuals in the IPIS Service group had a primary care physician (p = 0.037), whereas significantly more individuals in the PS group were using a rights advocacy program (p = 0.013).

In summary, individuals in the IPIS Service group were significantly older, more likely to live alone, and more frequently had a primary care physician than those in the PS group. In addition, 79.3% of the IPIS Service group were diagnosed with Alzheimer’s disease, and 56.2% were at CDR = 1, both of which were higher than the PS group. Furthermore, the IPIS Service group exhibited a significantly higher frequency of independence in BADL.

### 3.2 Objective outcomes: issues faced by the individuals from the two services

The results are shown in **Table 1**.

#### 3.2.1 Mental health issues

Overall, the frequency of mental health issues did not differ between the two groups. However, specific subcategories varied significantly.

The IPIS Service group had a significantly higher frequency of undetected dementia (p = 0.015) and undetected Behavioral and Psychological Symptoms of Dementia (BPSD) (p = 0.002). The PS group had a significantly higher frequency of undetected mental health conditions beyond dementia (p<0.001) and acute phases of mental health conditions (p<0.001).

#### 3.2.2 Physical health issues

The overall frequency of physical health issues did not differ between the two groups. However, when examining subcategories, none of the individuals in the IPIS Service group was assessed as having delirium, trouble with hospital staff, or issues related to end-of-life care. These issues were significantly more common in the PS group (p<0.001, p<0.001, p=0.013, respectively).

#### 3.2.3 Family issues

The overall frequency of family issues was significantly higher in the PS group (p < 0.001). Specifically, the PS group had significantly higher frequencies of Family Structure Issues (p = 0.048), Caregivers with Psychiatric Disorders (p < 0.001), and Abuse (p < 0.001).

#### 3.2.4 Issues of neighborhood communication

The overall frequency of neighborhood communication issues was significantly higher in the PS group (p = 0.008). Within this category, the frequency of severe domestic squalor was also significantly higher in the PS group (p = 0.035).

#### 3.2.5 Financial Issues

The overall frequency of financial issues, including the frequencies of the two subcategories, did not differ significantly between the two groups (p = 0.066).

### 3.3 Subjective outcomes: characteristics of cases for which the IPIS service was less equipped and another service would have been a more appropriate option

Currently, there are 19 IPISTs in the district, none of which include members with psychiatric service experience. All the dementia support doctors are primary care physicians. Focus group interviews (FGIs) were conducted with 17 of the 19 CGSCs, involving a total of 42 IPIST members. Eleven of 19 dementia support doctors and all three IPIST members from the local government participated in the interviews. For the Psychogeriatric Service (PS) team, two out of three geriatric psychiatrists and all three PS members from the local government participated in the interviews.

The number of members who indicated that cases falling into each subcategory listed in **Table 1** were difficult for the IPIS Service to manage was counted and is also presented in the same table.

The characteristics most frequently identified by participants as making the IPIS Service less equipped included: 1) undetected mental health conditions beyond dementia (86.1%) and the acute phase of mental health conditions (88.9%); 2) all subcategories under Category C: Family issues (52.8%~88.9%); 3) All subcategories under Category D: Issues related to neighborhood communication (52.8~55.6%); and 4) cases in which the patient was a victim of fraud (55.6%).

Based on the narrative data, IPIST members reported difficulties in distinguishing severe Behavioral and Psychological Symptoms of Dementia (BPSD) from symptoms of other mental health conditions. Furthermore, 8 of 11 dementia support doctors noted that such cases exceed the management capacity of primary care physicians. Furthermore, several “psychosocial issues” were identified by dementia support doctors as falling outside the scope of the IPIS Service’s responsibilities. These issues included:

Family issues: family structure with no potential caregiver (5 of 11); family caregiver has mental health conditions (4 of 11); family members interfering with service utilization (7 of 11); and abuse (7 of 11).

Neighborhood communication issues: aggressive behavior toward neighbors (5 of 11), exclusion from the community (5 of 11); severe domestic squalor (5 out of 11).

Financial issues arising from a background of poverty (4 of 11), and victim of fraud (6 of 11).

One dementia support doctor remarked that the IPIS Service is designed for individuals at risk of facing challenges, rather than those already experiencing significant difficulties. Similarly, a CGSC member noted that the IPIS Service tends to be less equipped for individuals whose difficulties have persisted for an extended period.

In summary, the IPIS Service was well-equipped for cases of mild dementia without severe BPSD that require early intervention. However, it was found less equipped in situations involving: 1) mental health conditions other than dementia; 2) long-standing issues such as family-related challenges; and 3) crises already in progress, including conflicts with third parties such as neighborhood disputes or incidents of fraud.

## 4 Discussion

As the global population ages and the number of PLWD increases, task-shifting has become an essential strategy in dementia care. In Japan, the task concerning PLWD is shifted to the IPIS Service, which is usually led by primary care physicians, from the psychogeriatric outreach service led by psychiatrists.

The present study highlights several challenges encountered in this task-shifting process. One major issue is the limited psychiatric expertise within IPIS teams, which complicates the management of older individuals presenting with mental health needs beyond dementia. Cases involving delirium, end-of-life care, or conflicts with medical professionals are frequently referred to the psychogeriatric service. In addition, psychosocial issues such as family conflicts and neighborhood disputes are also frequently referred to the psychogeriatric service.

A nationwide survey (22) reported that approximately 40% of cases handled by IPIS Service involved complex psychosocial care needs requiring significant psychiatric intervention. In this study, we define such individuals as “persons in complex and difficult situations with overlapping psychosocial challenges, including mental health issues, physical health issues, family problems, neighborhood communication difficulties, and financial concerns.” While multidisciplinary teams including primary care physicians can be well equipped for early-stage dementia interventions, they may be less equipped to support individuals already facing longstanding psychosocial or psychiatric difficulties.

Based on insights from the field, we propose a collaborative care model designed to strengthen the ecosystem of dementia outreach services:

Setting:

The IPIS Service functions as the primary outreach platform for older persons in the community.

Workforce structure:

The IPIS team consists of a dementia support doctor and professionals from Community General Support Centers (CGSCs), operating under local government leadership. The Psychogeriatric Service (PS) team includes a geriatric psychiatrist and municipal staff.

Collaborative mechanism:

When IPIS encounters difficulty in assessment or care planning, they can request consultation from the PS team. In this model, PS serves a role analogous to consultation-liaison psychiatry (CLS) in general hospitals.

Communication mechanism:

A standardized liaison sheet was developed to facilitate clear and effective information sharing between IPIS and PS teams, supporting coordinated care.

Triage support:

To promote timely access to appropriate services, a flowchart was developed to help CGSC members assess whether a case requires referral to the IPIS Service or PS, or whether it can be managed using standard procedures outlined in the manual (see **Supplementary Flowchart**).

Education and training:

Joint training sessions are held annually to enhance mutual understanding and strengthen inter-team collaboration.

Funding:

Both services are publicly funded. The IPIS Service is supported through national dementia policy, while PS is funded at the municipal level. Project-related costs also cover activities related to inter-service collaboration.

Currently, the task-shifting of outreach services for PLWD from psychiatrists to dementia support doctors has not been fully achieved. Two potential solutions are proposed: 1) establishing a formal consultation system between dementia support doctors and psychiatrists; and 2) providing further education for dementia support doctors to enable them to manage mental health conditions beyond dementia and address psychosocial challenges.

Over the past several decades, Consultation-Liaison Psychiatry Services (CLS) have expanded beyond traditional inpatient settings to primary care (23). Frost (24) proposed four post-diagnostic dementia care models delivered by primary care; 1) primary care physician- led; 2) primary care physician-led with specialist consulting support; 3) primary care physician-care manager partnership; and 4) integrated models. The collaborative model proposed by the present study, between the IPIS Service/primary care physicians and psychogeriatric services/psychiatrists, closely aligns with Frost’s second model: primary care physician-led with specialist consulting support. This approach could be considered as a form of community-based CLS, where psychiatric specialists provide consultation to non-psychiatric specialists, with IPIS/primary care physicians leading dementia care in the community.

Even with early intervention in dementia, pre-existing psychosocial issues may persist. Without sufficient clinical training in psychiatric care, it is understandable that primary care physicians serving as dementia support doctors may struggle to distinguish between BPSD and other mental health conditions, as well as to address psychosocial challenges. This exposes the limitations of the current task-shift approach and suggests that the study’s findings may have broader implications.

While differentiating service targets based on dementia helps address the specialist shortage, further collaboration between psychiatrist-led and primary care-led teams is essential for building an integrated care ecosystem.

This study has several limitations. First, due to the observational and exploratory nature of the research, the populations served by the IPIS and PS services differ significantly in age, functional status, and living conditions. These differences reflect the distinct intake criteria and organizational mandates of the two services, and were in fact one of the study’s key findings. As such, direct comparisons of patient-level outcomes between the two groups—without statistical adjustments such as propensity score matching or multivariate regression—are not appropriate.

Second, this study focuses on service processes and system-level characteristics rather than clinical or patient-centered outcomes. While we did not incorporate standardized outcome metrics, qualitative insights from service providers helped illuminate the contextual challenges and structural limitations of task-shifting in dementia outreach care.

Future research should build on these findings by incorporating validated outcome measures and applying statistical methods to adjust for baseline differences, thereby enabling more rigorous evaluation of effectiveness.

## 5 Conclusions

The shift of dementia care from specialist-led services to general practice, as part of Japan’s national dementia strategy, has introduced new challenges, particularly in managing complex psychiatric and psychosocial issues. The present study highlights the limitations of the IPIS Service, in which primary care physicians, often without psychiatric training, serve as the main medical leads. Although primary care physicians are crucial for early intervention and management of dementia, they are often less prepared to handle cases with significant psychiatric or psychosocial complexity.

To address these challenges, two strategies are proposed: 1) establishing a structured consultation system between general practitioners and psychiatrists, and 2) providing targeted education to enhance the capacity of primary care physicians to manage psychiatric conditions beyond dementia. A promising model is the primary care physician-led approach with specialist consulting support, which offers a collaborative framework wherein psychiatric specialists provide guidance on more complex cases.

Whereas task-shifting dementia care from psychiatrists to primary care physicians is an important step toward decentralizing dementia care, it must be supported by strong collaboration and educational initiatives. Ensuring that patients, particularly those with mental health and psychosocial challenges, receive comprehensive care is crucial. The insights from the present study contribute to the ongoing development of dementia care services in Japan and may inform similar efforts globally.

## Data Availability

The raw data supporting the conclusions of this article will be made available by the authors, without undue reservation.
