# Supplementary material for: Task-shifting in dementia care: a comparative analysis of consultation models and proposed collaborative ecosystem in Japan
Source: Front Psychiatry. 2025 Jun 13;16:1504753. doi: 10.3389/fpsyt.2025.1504753 (PMC12202652; doi:10.3389/fpsyt.2025.1504753)
Supplement: Supplementary file 1 [file DataSheet1.zip › Supplementary Table 1.pdf]

Table 1. Analytical frame of complex care needs of older persons, comparison of Psychogeriatric Service and Initial Phase Intensive Support Service, and number of participants who assessed the IPIS Service as ineffective for cases with the below characteristics

		Number of Cases with Care Needs per Category/Subcategory														Number of participants who indicated that cases with issues in that category were difficult for IPIS Service to manage.						
		PS Group		IPIS Service Group		Chi-squared	p -value			PS Group		IPIS Service Group		Chi-squared	p - value	Total		CGSCs	MSs of IPISTs	MSs of PS team	DSDs	Psys
		n	%	n	%					n	%	n	%			n	%	n	n	n	n	n
	Total number	121		213						121		213				36		17	3	3	11	2
Category								Subcategory														
A	Mental Health Issues	94	77.7%	179	84.0%	2.0	0.153	A1	Undetected dementia	68	56.2%	148	69.5%	5.9	0.015 *	1	27.8%	0	0	0	1	0
								A2	Untreated BPSD	16	13.2%	58	27.2%	9.3	0.002 *	15	41.7%	4	3	0	8	0
								A3	Undetected mental health conditions beyond dementia	24	19.8%	11	5.2%	16.9	<0.001 *	31	86.1%	15	6	0	8	2
								A4	Acute phase of mental health conditions	6	5.0%	0	0.0%	12.4	<0.001 *	32	88.9%	15	6	0	9	2
B	Physical Health Issues	44	36.4%	62	29.1%	1.9	0.173	B1	Neglect of physical state	28	23.1%	62	29.1%	1.4	0.234	10	27.8%	8	0	1	1	0
								B2	Delirium	12	9.9%	0	0.0%	19.1	<0.001 *	1	2.8%	1	0		0	0
								B3	Having trouble with hospital staff	18	14.9%	0	0.0%	38.4	<0.001 *	16	44.4%	4	3	1	6	2
								B4	Issues related to end-of-life care	3	2.5%	0	0.0%	6.1	0.013 *	8	22.2%	8	0		0	0
C	Family Issues	93	76.9%	88	41.3%	40.9	<0.001 *	C1	Family structure with no potential caregiver	45	37.2%	57	26.8%	3.9	0.048 *	19	52.8%	10	3	1	5	0
								C2	Caregiver has mental health condition	35	28.9%	28	13.1%	12.1	<0.001 *	21	58.3%	10	3	3	4	1
								C3	Interference with service use by family members	10	8.3%	13	6.1%	0.6	0.459	20	55.6%	9	3	1	7	0
								C4	Abuse	36	29.8%	0	0.0%	81.0	<0.001 *	32	88.9%	17	3	3	7	2
D	Issues of Neighborhood Communication	47	38.8%	53	24.9%	7.0	0.008 *	D1	Aggressive behavior towards neighbors	8	6.6%	8	3.8%	1.3	0.249	20	55.6%	12	3	0	5	0
								D2	Exclusion from the community	30	24.8%	36	16.9%	3.0	0.085	21	58.3%	13	3	0	5	0
								D3	Severe domestic squalor	24	19.8%	24	11.3%	4.5	0.035 *	19	52.8%	13	0	1	5	0
E	Financial Issues	32	26.4%	38	17.8%	3.4	0.066	E1	Money trouble	21	17.4%	24	11.3%	2.4	0.122	13	36.1%	8	0	1	4	0
								E2	Victim of fraud	11	9.1%	15	7.0%	0.4	0.506	20	55.6%	9	3	1	6	1

Abbreviations: PS, Psychogeriatric Service; IPIS Service, Initial-phase Intensive Support Service for Dementia; IPIST, Initial-phase Intensive Support Team; BPSD, Behavioral and Psychological Symptoms of Dementia; CGSC, Community General Support Center; MS, municipal staff; DSD, dementia support physician; Psy, geriatric psychiatrist
