# Supplementary material for: Task-shifting in dementia care: a comparative analysis of consultation models and proposed collaborative ecosystem in Japan
Source: Front Psychiatry. 2025 Jun 13;16:1504753. doi: 10.3389/fpsyt.2025.1504753 (PMC12202652; doi:10.3389/fpsyt.2025.1504753)
Supplement: Supplementary file 1 [file DataSheet1.zip › Supplementary Flowchart.pdf]

Quick Reference for Selecting Support Pathways (IPIS / PS / Standard Manual)

This chart is designed to support members of Community General Support Centers in assessing whether a case requires the use of the Initial Phase Intensive Support (IPIS) Service or the Psychogeriatric Service (PS), or whether it can be managed using standard procedures outlined in the ordinary manual.

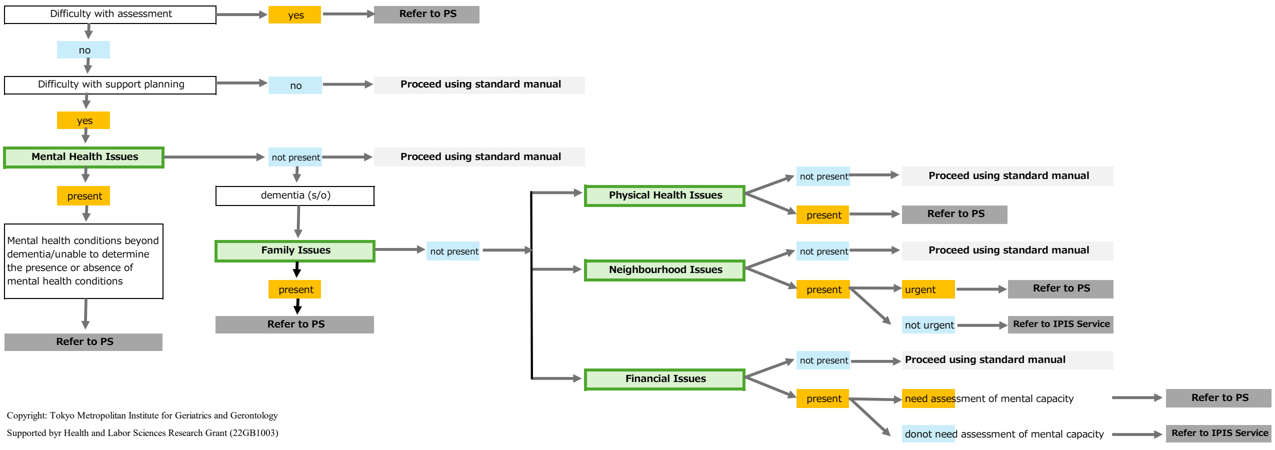

Copyright: Tokyo Metropolitan Institute for Geriatrics and Gerontology

Supported byr Health and Labor Sciences Research Grant (22GB1003)
