# Supplementary figures and images for: Task-shifting in dementia care: a comparative analysis of consultation models and proposed collaborative ecosystem in Japan
Source: Front Psychiatry. 2025 Jun 13;16:1504753. doi: 10.3389/fpsyt.2025.1504753 (PMC12202652; doi:10.3389/fpsyt.2025.1504753)

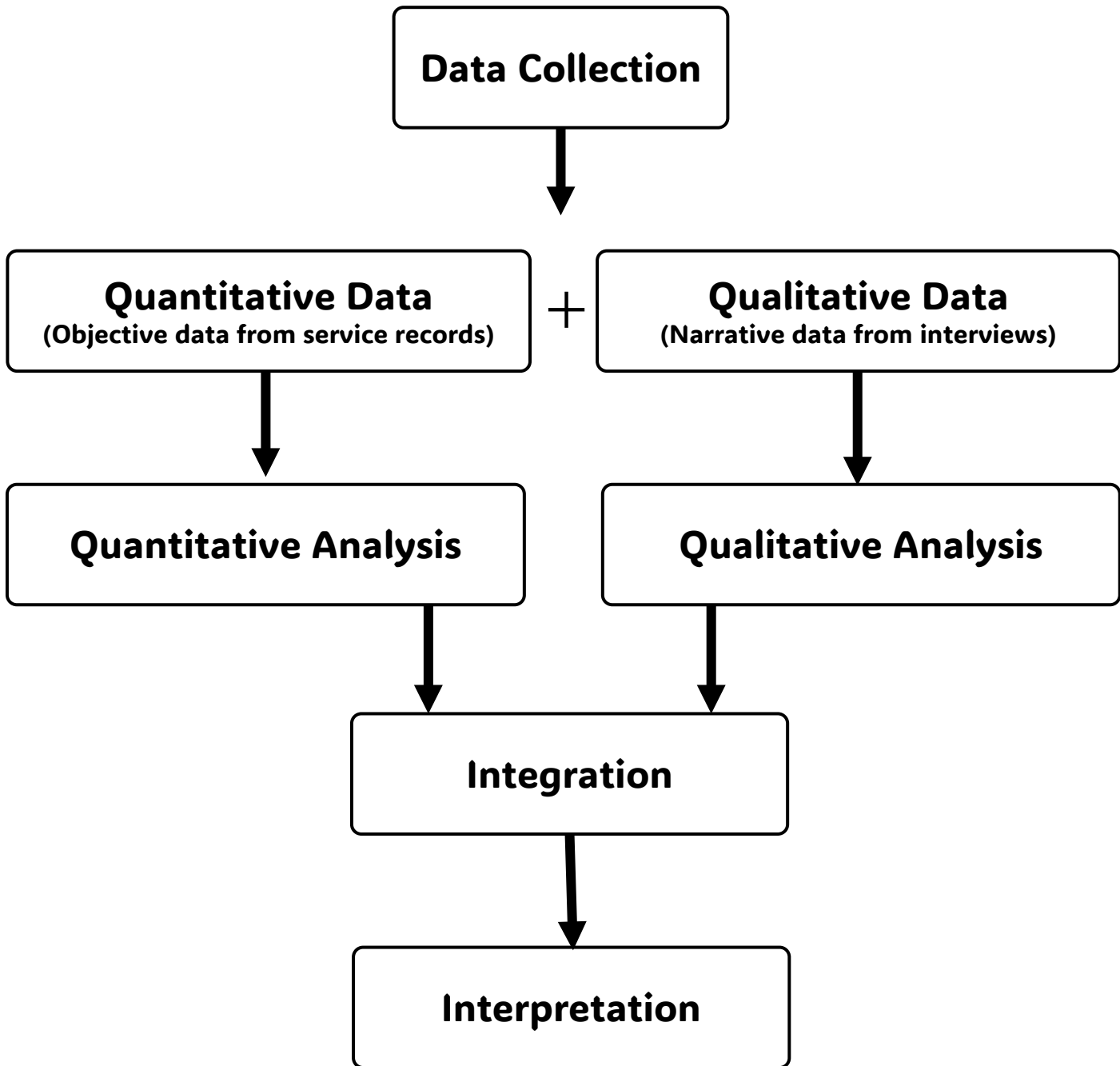

Supplement: Supplementary file 1 [file DataSheet1.zip › Supplementary Figure 1.pdf]
