# Supplementary material for: Task-shifting in dementia care: a comparative analysis of consultation models and proposed collaborative ecosystem in Japan
Source: Front Psychiatry. 2025 Jun 13;16:1504753. doi: 10.3389/fpsyt.2025.1504753 (PMC12202652; doi:10.3389/fpsyt.2025.1504753)
Supplement: Supplementary file 1 [file Supplementary Table 2.pdf › Supplementary Table 2.pdf]

Table 2. Description of the study population

		PS Group		IPIS Service Group			
		n	%	n	%	t<br>Chi-squared	p-value
		121		213			
**Sociodemographic characteristics**							
Age (y)	Mean ± SD	79.2 ± 7.4		81.6 ± 6.2		-3.2	< 0.001 *
Sex	Male	51	42.2%	76	35.7%		
	Female	70	57.9%	137	64.3%	1.4	0.243
Marital status	Have been married	90	74.4%	170	83.7%	4.1	0.043
	Never married	31	25.6%	33	16.3%		
	Currently single	87	71.9%	144	69.2%	1.1	0.571
Living condition	Living alone	61	50.4%	119	55.9%	17.9	<0.001 *
	Not living alone	59	49.2%	39	24.7%		
Source of income	Old age pensions	104	86.0%	148	69.5%	39.2	<0.001 *
	Welfare	12	9.9%	22	10.3%		
	No income	5	4.1%	4	1.9%		
Years of education	≤9	15	28.8%	13	23.6%	0.6	0.748
	10-12	23	44.2%	24	43.6%		
	≥13	14	26.9%	18	32.7%		
**Service utilization**							
Diagnosed psychiatric disorder (dementia) (+)		26(20)	21.5%	44(44)	20.7%	0.0	0.858
Have a primary care physician		59	48.8%	129	60.6%	4.4	0.037 *
Long-term care insurance services utilization (+)		34	28.1%	47	22.1%	1.5	0.219
Rights advocacy program (+)		3	2.5%	0	0.0%	6.1	0.013 *

	PS Group		IPIS Service Group			
	n	%	n	%	Chi-squared	p-value
	121		213			
**Clinical assessments**						
Clinical diagnosis of psychiatric disorders					87.7	<0.001 *
Mild Cognitive Disorder	16	13.2%	2	1.8%		
Dementia						
AD	37	30.6%	88	79.3%		
AD with CVD	16	13.2%	1	0.9%		
VaD	13	10.7%	2	1.8%		
Other types of dementia	5	4.1%	13	11.7%		
Other psychiatric disorder	29	24.0%	5	4.5%		
No psychiatric disorder	5	4.1%	0	0.0%		
Clinical Dementia Rating (CDR)					23.8	<0.001 *
0	4	3.3%	6	3.0%		
0.5	26	21.5%	12	6.0%		
1	53	43.8%	113	56.2%		
2	35	28.9%	70	34.8%		
3	3	2.5%	0	0.0%		
Basic activities of daily living (BADL)					16.1	<0.001 *
Independent	43	36.5%	116	54.5%		
Requires partial assistance	75	62.0%	97	45.5%		
Requires full assistance	3	2.5%	0	0.0%		

Abbreviations: SD, standard deviation; AD, Alzheimer's Disease; AD with CVD, Alzheimer's Disease with cerebrovascular disease; VaD, Vascular dementia
